# Neutral Ceramidase Is Required for the Reproduction of Brown Planthopper, *Nilaparvata lugens* (Stål)

**DOI:** 10.3389/fphys.2021.629532

**Published:** 2021-02-24

**Authors:** Xiao-Xiao Shi, Mu-Fei Zhu, Ni Wang, Yuan-Jie Huang, Min-Jing Zhang, Chao Zhang, Soomro A. Ali, Wen-Wu Zhou, Chuanxi Zhang, Cungui Mao, Zeng-Rong Zhu

**Affiliations:** ^1^State Key Laboratory of Rice Biology, Key Laboratory of Molecular Biology of Crop Pathogens and Insects, Ministry of Agriculture, Institute of Insect Sciences, Zhejiang University, Hangzhou, China; ^2^People’s Government of Fenshui Town, Tonglu County, Hangzhou, China; ^3^Department of Medicine and Stony Brook Cancer Center, The State University of New York at Stony Brook, Stony Brook, NY, United States; ^4^Hainan Research Institute, Zhejiang University, Sanya, China

**Keywords:** neutral ceramidase, brown planthopper, reproduction, apoptosis, ceramide

## Abstract

Ceramides are bioactive sphingolipids that have been implicated in insect development; however, their role in insect reproduction remains poorly understood. Here, we report the pivotal role of neutral ceramidase (NCER) in the female reproduction of the brown planthopper (BPH), *Nilaparvata lugens* (Stål), a significant pest in rice cultivation in Asia. LC-MS/MS demonstrated that, among different developmental stages of BPH, the levels of ceramides were highest in 1st instar nymphs and lowest in adults. The transcription of *NCER* was negatively correlated with the levels of ceramides at different developmental stages of BPH, in that the transcript levels of *NCER* were the highest, whereas ceramides levels were the lowest in BPH adults. Knocking down *NCER* through RNA interference (RNAi) increased the levels of ceramides in BPH females and ovaries, which resulted in a delay in oocyte maturation, a reduction in oviposition and egg hatching rate, as well as the production of vulnerable offspring. Transmission electron microscopy (TEM) analysis and TdT-mediated dUTP Nick-End Labeling (TUNEL) assays showed mitochondrial deficiency and apoptosis in *NCER*-deficient oocytes. Taken together, these results suggest that NCER plays a crucial role in female reproduction in BPH, likely by regulating the levels of ceramides.

## Introduction

The brown planthopper (BPH), *Nilaparvata lugens* (Stål), one of the most economically devastating rice pests in Asia, causes severe agricultural damages by sucking plant juice and spreading plant viruses. Since a mature female can lay hundreds of eggs, the BPH population can expand rapidly, damaging rice yield; therefore, there is an urgent demand to control BPH (Dong et al., 2011). The management of BPH is mostly achieved using pesticides, which contaminates the environment and induces food insecurity. Identifying the internal signaling pathways that regulate BPH development and reproduction will facilitate novel approaches for containing the outbreak of BPH.

Sphingolipids are not only the structural components of cellular membranes, but they also have roles in the signaling pathways involved in cell proliferation, differentiation, and apoptosis of various species (Hannun, 1996; Prieschl and Baumruker, 2000). Emerging evidence indicates that ceramides, a type of sphingolipid, are located in the central hub of the sphingolipid metabolic network and have a role in regulating the development and reproduction of mammals and insects. Ceramides are present in the human placenta and are increased in the placental villous trophoblasts of insulin-treated gestational diabetes mellitus patients (Mejia et al., 2019). High levels of ceramides were also detected in pregnancies complicated by preeclampsia (Melland-Smith et al., 2015). Ceramides and their derivatives, sphingosine-1-phosphates (S1P), can synergistically as well as antagonistically mediate embryonic stem (ES) cell differentiation in mice (Wang et al., 2018). The perturbation in ceramide metabolic homeostasis committed by gene absence triggered the developmental defects in *Drosophila* (Kraut, 2011). Ceramides and S1Ps regulated the reproductive development of *Drosophila* (Phan et al., 2007). The *Drosophila* Sply^05091^ mutants had severely abnormal flight muscles and excessive apoptosis in their reproductive organs, resulting in supernumerary spermathecae, degenerative ovaries, and severely reduced testes (Herr et al., 2003; Phan et al., 2007). In addition, mutations in the serine palmitoyl-transferase (SPT) enzyme subunit of *Drosophila* resulted in defective synaptic structure and function (West et al., 2018).

**FIGURE 1:**
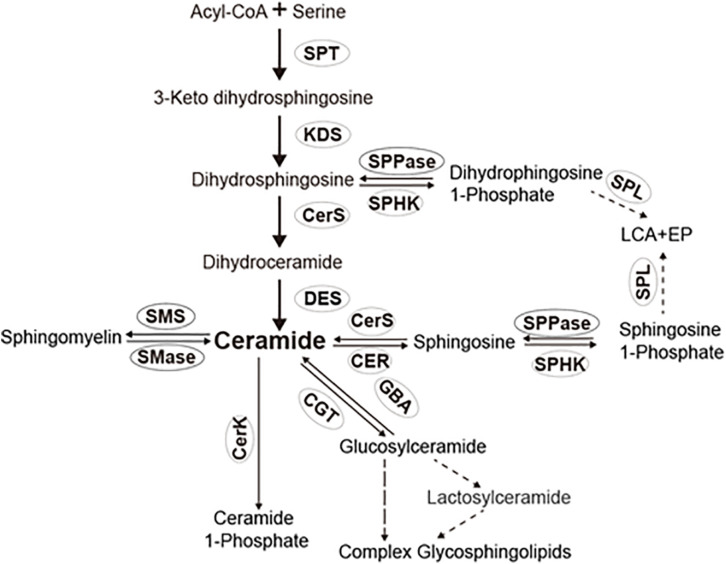
Sphingolipid pathway in insects. *SPT*, serine palmitoyl transferase; *KDS*, 3-dehydrosphinganine reductase; *CerS*, ceramide synthases; *CER*, ceramidase; *SPHK*, sphingosine kinase; *CerK*, ceramide kinase; *SPL*, sphingosine-1-phosphate lyase; *SPPase*, sphingosine-1-phosphate phosphatase; *SMS*, sphingomyelin synthase and/or ceramide choline phosphotransferases; *SMase*, sphingomylinases; *CGT*, ceramide glucotransference; and *GBA*, glucosyl ceramidase.

Ceramides are generated mainly via the *de novo* pathway, salvage pathway, and sphingomyelin pathway (Figure 1). In the *de novo* pathway, ceramide synthesis is carried out sequentially by SPT, 3-dehydrosphinganine reductase, (dihydro) ceramide synthase, and dihydroceramide desaturase (Figure 1). In the salvage pathway, glycosphingolipids are broken down into sphingosine in the lysosomes, and sphingosine is subsequently acylated with various acyl chains to form ceramides. In the sphingomyelin pathway, sphingomyelins are converted to ceramides by sphingomyelinases (Hannun et al., 2001; Hannun and Obeid, 2011; Kraut, 2011; Mencarelli and Martinez Martinez, 2013). Once generated in cells, ceramides are hydrolyzed into free fatty acids and sphingosine by ceramidases (Mao and Obeid, 2008). The enzymes responsible for ceramide metabolism are relatively conserved across different species (Hannun and Obeid, 2011). However, *Drosophila* is traditionally described as lacking sphingomyelins and its metabolic enzyme sphingomyelin synthase (Acharya and Acharya, 2005). Unlike *Drosophila*, BPH has sphingomyelins and orthologs of sphingomyelin synthase (SMSL1 and SMSL2) (Shi et al., 2019). The above findings indicated the peculiar differences between insects in the structure, and metabolism of sphingolipids. While the sphingolipid metabolic enzymes and their orthologs in *Drosophila* are extensively studied (Acharya and Acharya, 2005), the ceramide metabolic enzymes in other insects remain ambiguous.

Ceramidases (CDases) are enzymes that hydrolyze ceramides into sphingosines and free fatty acids. Based on the optimal pH for their catalytic activity, CDases are classified into three groups, acid ceramidase (AC), neutral ceramidase (NCER), and alkaline ceramidase (ACER). Five human ceramidases, including acid ceramidase (AC/ASHA1), neutral ceramidase (NC/ASAH2), alkaline ceramidase 1 (ACER1), alkaline ceramidase 2 (ACER2), and alkaline ceramidase 3 (ACER3), encoded by five different genes, have been identified (Coant et al., 2017). Acid, neutral, and alkaline ceramidases have been identified in insects (Yoshimura et al., 2002; Monjusho et al., 2003; Yang et al., 2010; Zhou et al., 2011; Shi et al., 2018).

A growing body of work has proposed cell fate to be dependent, in part, on the dynamic balance of ceramides that were catalyzed by CDases. The disorder of ceramide metabolism induced by the dysfunction of different CDases caused many pathological defects (Li et al., 2002; Xu et al., 2006). For instance, an *ASAH1* mutation causes Farber disease (FD), a lysosomal storage disorder (Li et al., 2002). The same mutation causes early embryonic lethality in mice homozygotes and progressive lipid storage disease in mouse heterozygotes (Li et al., 2002). Also, the downregulation of *ACER2* (*haCER2*) committed by RNA interference (RNAi) enhances the serum deprivation-induced growth arrest and apoptosis of HeLa cells (Xu et al., 2006). In addition, the inactivation of alkaline ceramidase (*Dacer*) prolongs the development and lifespan of *Drosophila melanogaster* (Yang et al., 2010).

However, CDases have been less extensively studied in insects rather than mammals (Hannun and Obeid, 2011; Kraut, 2011; Mencarelli and Martinez Martinez, 2013). The enzymatic properties of the CDases in *Tribolium* (Zhou et al., 2011), *Laodelphax striatellus* (Zhou et al., 2013), and *N. lugens* (Shi et al., 2018) have been characterized, but their biological roles remain poorly understood. Our previous study showed that NCER, the neutral CDase of *N. lugens* (NlnCDase), have a higher transcript levels and activity in adults and reproductive organs (ovaries and spermaries) (Shi et al., 2018); however, NlnCDase’s biological functions in adults or tissue development are still unknown. In this study, we have demonstrated that NCER plays a crucial role in regulating the development and function of BPH’s reproductive system by controlling ceramide metabolism in the ovaries.

## Materials and Methods

### Maintenance of Insects

The laboratory strain of *N. lugens*, which originated from a field population in the Huajiachi campus of Zhejiang University, Hangzhou, China, was reared at 27 ± 1°C with 70% relative humidity (RH) and under a 16-h light/8-h dark cycle. The wild-type and genetically modified BPH were maintained on the susceptible rice seedlings cv. Taichung Native 1 (TN1) as described previously (Shi et al., 2018).

### Sphingolipid Analysis

We collected the newly emerged 1st to 5th instar, female and male BPHs with a fresh mass of approximately 0.15 g per sample. Sphingolipids were extracted according to Bielawski’s method (Bielawski et al., 2009), and 10 ng of C_12_-ceramide was added as an internal standard. A 10 μl sample was separated on a reversed-phase analytical column (Agilent ZORBAX sb-C8, 2.1 × 150 mm, 3.5 um) with a flow rate of 0.3 ml/min at 35°C and analyzed with the MS/MS method. Solvent A was 2 mM of NH4AC in 0.1% HCOOH, and solvent B was methanol. The gradient was increased from 1 to 99% of solvent B over 20 min, 99 to 100% in 15 min, and held at 100% for the last 7 min. The total monitor time was set to 42 min. The sphingolipids in BPH with a knockdown of *NCER* were analyzed. At 0.1 g of fresh weight, BPH ovaries were dissected from 3-day-old females after dsRNA-*NCER* and dsRNA-*GFP* injection. The sphingolipids were extracted and quantified as mentioned above in triplicate experiments.

### RNA Interference of *NCER*

The primers were designed (Supplementary Table 1) and used to amplify fragments of *NCER* (XM_022339603.1) and *GFP* (ABE28520). The dsRNA-*NCER* and dsRNA-*GFP* were prepared as previously described (Shi et al., 2018). A dose of 0.1 μg of dsRNA-*NCER* was injected into the thorax, between the mesocoxa and the hindcoxa, of each 3-day-old 5th instar individual. The same dose of dsRNA-*GFP* was injected as the control. The transcript of *NCER* at 24 and 48 h post dsRNA injection was measured by qPCR to evaluate the efficiency of gene silencing. RNA extraction and qPCR quantification were performed as previously described (Shi et al., 2018).

### Bioassay of BPH

The role of NCER in BPH fitness was investigated by measuring the longevity, the number of eggs laid per mature female, hatchability, and the survival rate of the newly hatched nymphs after gene-silencing. We injected dsRNAs into 3-day-old 5th instar nymphs and the females that newly emerged 24 and 48 h after dsRNA injection were separately collected and reared at 27°C with 90% RH and a 16-h light/8-h dark cycle for further experiments. The female adults were individually transferred into glass tubes, 45 cm in depth and 5.5 cm in diameter, containing 1-month-old TN1 rice seedlings. Two wild-type BPH males were transferred into each tube for mating. After 7 days, all the BPH adults were removed from the tubes. Half of the rice seedlings were dissected to count the number of eggs laid by each female. The remaining rice seedlings were reared for seven more days to check egg hatchability. The survival of the nymphs produced by the dsRNA-injected females was counted to determine the effect of *NCER* on the fitness of offspring.

### Ovary Observation

Brown planthopper female adults were separately collected on the 1st, 3rd, and 5th day after dsRNA-*NCER* or dsRNA-*GFP* injection. The ovaries from the two groups were obtained, stored in phosphate-buffered saline (PBS), and photographed under a Nikon SMZ 745T stereomicroscope with a digital camera to observe the dynamic development of the BPH ovaries.

### TUNEL Assay

Brown planthopper ovaries were separately collected on the 1st, 3rd, and 5th day post dsRNA injection. The DNA fragmentation in BPH ovarian cells was evaluated with the DeadEnd^TM^ Fluorometric TdT-mediated dUTP Nick-End Labeling (TUNEL) System (Promega) according to the manufacturer’s instructions. The fluorescein-12-dUTP provided in the kit was used to label the broken DNA in apoptotic cells. The nuclei were counterstained with a 4′,6-diamidino-2-phenylindole (DAPI) solution (Beyotime Biotechnology). Apoptotic cells labeled positively by fluorescein-12-dUTP (green fluorescence) and total cells with a DAPI staining (blue fluorescence) were imaged under a Zeiss LSM780 confocal laser scanning microscope (Zeiss, Göttingen, Germany).

### Transmission Electron Microscopy

Brown planthopper ovaries were separately collected on the 1st, 3rd, and 5th day post dsRNA injections and prepared according to the transmission electron microscopy (TEM) examination procedures. The BPH oocytes were analyzed with TEM to visualize the structural differences in cellular organelles after dsRNA injection.

### Statistical Analysis

Pearson’s correlation analysis of the levels of *NCER* transcript and ceramides in BPH at different developmental stages was conducted by the data processing system (DPS) 13.5 (Tang and Zhang, 2013). Pearson’s correlation coefficient (|r|) between 0.6 and 0.8 indicated a significant correlation. qRT-PCR results were calculated according to the 2^–ΔΔCt^ method of normalized relative quantification. The differences between groups were compared using the Student’s *t*-test. *P* values of less than 0.05 were considered statistically significant. The least significant difference (LSD) method of multiple comparisons of parental and control groups was applied when the probability for ANOVA was statistically significant.

## Results

### Female BPH Had the Lowest Level of Ceramides

**FIGURE 2:**
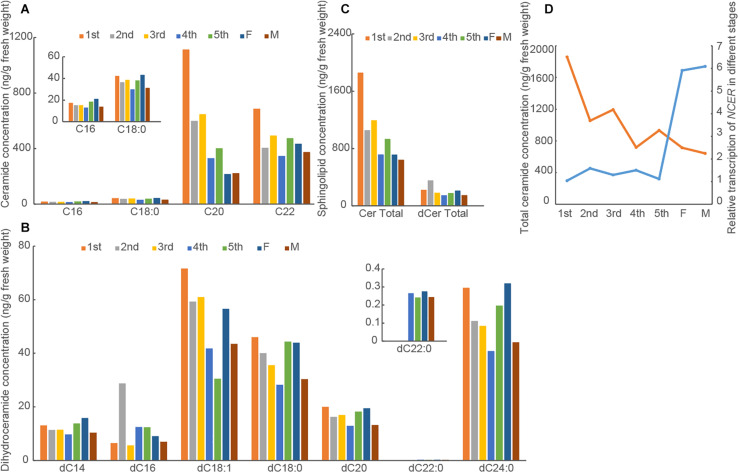
Levels of ceramides and dihydroceramides vary in BPH at different developmental stages. BPH at indicated developmental stages were subjected to measurements of the concentrations of ceramides **(A)** and dihydroceramides **(B)**. The data were expressed as mean of two replications. **(C)** The total ceramide concentrations (Cer Total) and total dihydroceramide concentrations (dCer Total) were caculated. **(D)** The correlation analysis between *NCER* transcript levels (blue line) and total ceramide concentrations (orange line) in BPH at different developmental stages. The data of *NCER* transcription were expressed as mean ± SE (*n* = 3). 1st, the first instar nymph; 2nd, the second instar nymph; 3rd, the third instar nymph; 4th, the fourth instar nymph; 5th, the fifth instar nymph; F, Female; M, Male.

Before we investigated the role of sphingolipids for insect development, we first determined the levels of ceramides and dihydroceramides in BPH at different developmental stages. With LC-MS/MS, we demonstrated that C20:0 (d18:1/C20) and C22:0 (C18:1/C22) ceramides were more abundant than C16:0 (d18:1/C16:0) and C18:0 (C18:1/C18:0) ceramides among the sphingosine-containing ceramides. Meanwhile, C18:0 (d18:0/C18:0), C24:0 (d18:0/C24:0), and C18:1 (d18:0/C18:1) dihydroceramides were more abundant than C14:0 (d18:0/C14:0), C16:0 (d18:0/C16:0), C20:0 (d18:0/C20:0), and C22:0 (d18:0/C20:0) dihydroceramides among the dihydrosphingosine-containing ceramides across the developmental stages (Figures 2A,B). We found that the major individual ceramide species and total ceramides were more abundant than the major individual dihydroceramide species and total dihdyroceramides across the developmental stages (Figure 2C). The C20:0 (d18:1/C20) and C22:0 (C18:1/C22) ceramide species and total ceramides were least abundant in the adult females and males but most abundant in the 1st instar nymphs. In contrast, the level of dihydroceramide species irregularly varied across the developmental stages. These results suggested that the levels of ceramides decreased from the 1st instar nymphs to adults. Since we previously showed NCER’s role in the hydrolysis of ceramides into sphingosine and free fatty acid and studied the expression pattern of *NCER* (*NlnCDase*) in BPH different developmental stages (Shi et al., 2018), we hypothesized that *NCER* expression might correlate with the low levels of total ceramides in BPH adults. Indeed, Pearson’s correlation analysis showed a negative relationship between the levels of total ceramides and the levels of *NCER* transcripts (*P* < 0.05, *r* = −0.74) (Figure 2D).

### NCER Is Essential for BPH’s Ovary Development, Fecundity, and Offspring Fitness

**FIGURE 3:**
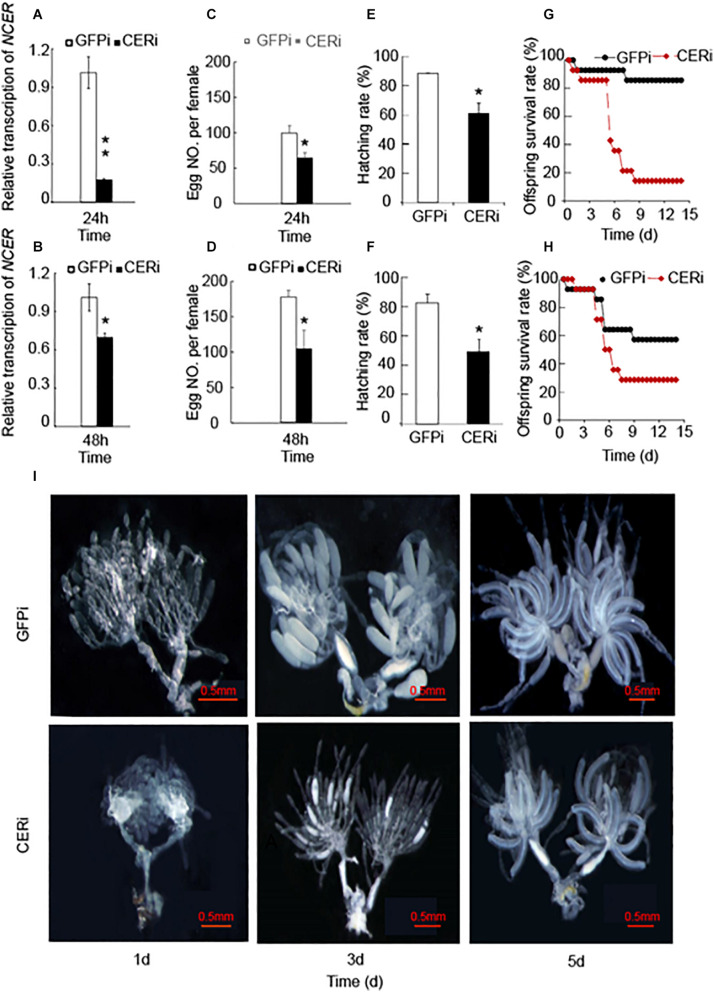
*NCER* knockdown reduces female BPH fecundity. The 5th instar nymphs were injected with dsRNA of *NCER* or *GFP*. CERi, dsRNA-*NCER* injected; GFPi, dsRNA-*GFP* injected. The BPH females that emerged in 24 h **(A,C,E,G)** or 48 h **(B,D,F,H)** after dsRNA injection were utilized for the bioassay experiments. **(A,B)** The RNAi effenciency after dsRNA injection in BPH females. **(C,D)** The number of eggs laid by each female in seven days. **(E,F)** The hatching rate of eggs laid by BPH female. **(G,H)** The survival rates of offspring produced by BPH female. **(I)** The developmental status of BPH ovaries. BPH ovaries were dissected from 1, 3, 5 days-old females for the photography. The data was expressed as mean ± SE (*N* = 3). Significance is shown by “*” at the *P* < 0.05 and “**” at the *P* < 0.01 level.

Given *NCER*’s high expression in the BPH ovaries (Shi et al., 2018), we investigated the role of NCER in female reproductive development. We knocked down *NCER* expression in female adults by RNAi. qPCR analysis confirmed that the females emerging from the dsRNA-*NCER*-injected (CERi) nymphs had lower levels of *NCER* transcripts than the females emerging from the dsRNA-*GFP*-injected (GFPi) nymphs (Figures 3A,B). The RNAi suppressed *NCER* expression could be significantly sustained for 24 h (Figure 3A) and 48 h (Figure 3B) after dsRNA injection. Thus, the BPH females which newly emerged 24 h or 48 h after the dsRNA injection were assayed for oviposition, longevity, egg hatchability, and offspring’s viability. The CERi females laid significantly fewer eggs (Figures 3C,D) and had a lower egg hatchability (Figures 3E,F) than the GFPi females. The survival rates of the CERi females’ offspring were reduced compared to the offspring produced by GFPi females (Figures 3G,H). Moreover, the degree of *NCER* suppression correlated with the severity of the reproductive defects (Figures 3A–H). These results suggest that NCER plays a role in maintaining female fecundity and offspring fitness.

Given the reduction of female fecundity by the knockdown of *NCER*, we investigated whether NCER was necessary for BPH’s ovarian development and maturation. The ovaries of the CERi or GFPi females were analyzed. The CERi females had fewer ovarioles than the GFPi females (Figure 3I). Oogenesis formation was also inhibited, and ovary development became asynchronized in the CERi females compared to the GFPi females on the 3rd day after emergence (Figure 3I). The decrease of the fecundity of BPH could be explained by the arrest of ovarian development in BPH due to the suppression of *NCER*.

### *NCER* Knockdown Accelerates Apoptosis Through Damage to the Cytoplasmic Membrane, Nuclei, and Mitochondria in BPH Oocytes

**FIGURE 4:**
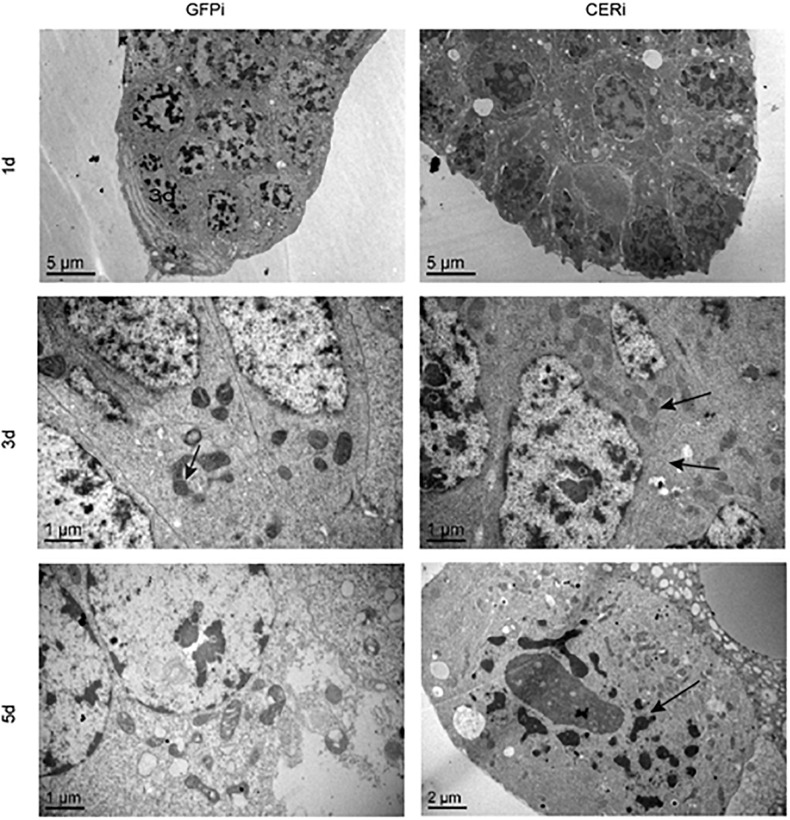
*NCER* knockdown induces apoptosis in BPH oocytes. Ovaries were collected from GFPi or CERi females at 1, 3, or 5 days post injection with dsRNA and subjected to transmission electron microscopic (TEM) analysis. The difference of cellular structure was pointed by black arrow.

We performed TEM and TUNEL analysis in GFPi and CERi ovaries to investigate how *NCER* knockdown affected female fecundity and ovary development in BPH. TEM analysis showed that *NCER* knockdown destroyed the plasma membrane and decondensed the nuclei (arrow; Figure 4). Although there were more mitochondria in CERi oocytes than GFPi oocytes, the structure of mitochondrial cristae in CERi oocytes was damaged (Figure 4). These results suggest that *NCER* knockdown causes the dysfunction of the cytoplasmic membrane, nuclei, and mitochondria, inducing the apoptosis of oocytes. We observed the apoptotic process in the BPH ovaries at different time points with the fluorometric TUNEL assay. Nicked DNA was detected in CERi and GFPi oocytes at the 3rd and 5th day post dsRNA injection, respectively (Supplementary Figure 1). These data suggest that *NCER* knockdown accelerates the apoptosis of oocytes.

### *NCER* Knockdown Increases the Levels of Ceramides and Dihydroceramides in the Ovary

**FIGURE 5:**
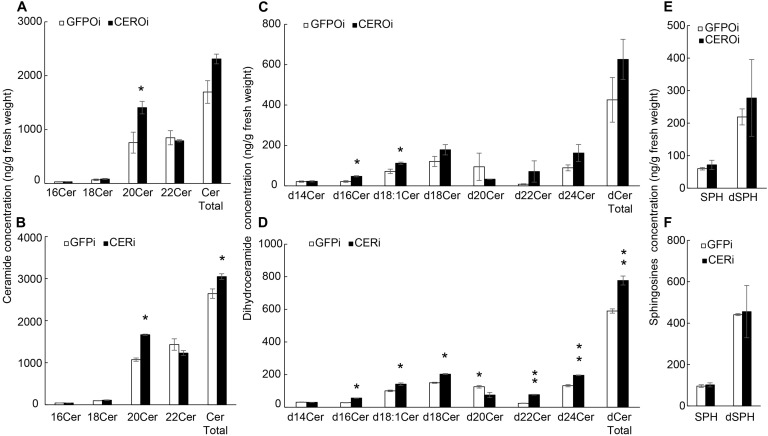
*NCER* knockdown increases the levels of (dihydro)ceramides without affecting the levels of sphingoid bases. Ovaries were collected from GFPi or CERi females at 3rd day post injection with dsRNA injection and subjected to LC-MS/MS for the levels of ceramides in BPH ovaries **(A)** or BPH bodies **(B)**; the levels of dihydroceramides in BPH ovaries **(C)** or in BPH bodies **(D)**; the levels of sphingosine and dihydrosphingosine in BPH ovaries **(E)** or in BPH bodies **(F)**. CERi, BPH bodies with dsRNA-*NCER* injected. GFPi, BPH bodies with dsRNA-*GFP* injected. CEROi, BPH ovaries with dsRNA-*NCER* injected. GFPOi, BPH ovaries dsRNA-*GFP* injected. The data was expressed as mean ± SE (*N* = 3). Significance is shown by “*” at the *P* < 0.05 and “**” at the *P* < 0.01 level.

Ceramides and sphingosines were quantified in the ovaries and whole body of GFPi or CERi female adults to explore how *NCER* knockdown caused apoptosis in BPH oocytes. LC-MS/MS showed that *NCER* knockdown increased the levels of the C20:0 (d18:1/C20) ceramide (Figure 5A), and C16:0 (d18:0/C16:0) and C18:1 (d18:0/C18:1) dihydroceramides in BPH ovaries (Figure 5C). Also, the levels of the C20:0 (d18:1/C20) ceramide (Figure 5B), C16:0 (d18:0/C16:0), C18:1 (C18:0/C18:1), C18:0 (C18:0/C18:0), C22:0 (d18:0/C22:0), and C24:0 (d18:0/C24:0) dihydroceramides (Figure 5D) were increased by the *NCER* knockdown in the BPH whole body. Contrary to other dihydroceramides, the levels of the C20:0 (d18:0/C20:0) dihydroceramide in the whole body decreased after the inhibition of *NCER* (Figure 5D). *NCER* knockdown significantly increased the total ceramides (Figure 5B) and total dihydroceramides (Figure 5D) in the BPH whole body. However, *NCER* knockdown did not affect the levels of sphingoid bases (sphingosine and dihydrosphingosine) in ovaries (Figure 5E) or whole bodies (Figure 5F). These results suggest that NCER plays an important role in ceramide homeostasis in the BPH ovary.

## Discussion

This study demonstrates that the levels of ceramides in BPH are decreased, whereas the transcript level of *NCER*, whose encoded protein catabolizes ceramides, is increased during BPH development from nymphs to adults. Our data suggest that NCER plays a crucial role in preventing the premature degeneration of the BPH ovary by maintaining low levels of ceramides in the ovary.

Ceramides are precursors of complex sphingolipids, such as sphingomyelins and glycosphingolipids. These sphingolipids serve as the critical structural components of cell membranes. Therefore, high levels of ceramides are essential to constructing the cellular membranes of the nymphs. Since ceramides are pro-death bioactive lipids, their aberrant accumulation may lead to unregulated cell death and tissue atrophy. Indeed, there is ample evidence suggesting that increased ceramides are associated with neurodegenerative disorders, such as Alzheimer’s, Parkinson’s, and Huntington’s diseases (Czubowicz et al., 2019). Our study showed that BPH lowers the level of ceramides in tissue by expressing *NCER* to break down the ceramides. As ceramides have been shown to induce growth arrest, the levels of ceramides must be suppressed in tissues with active cell division. Indeed, we demonstrate for the first time that ceramides are maintained at lower levels in the female ovary, whose cells are highly proliferative.

The generation and clearance of ceramide are regulated by complex and integrated metabolic pathways involving several specialized enzymes (Hannun et al., 2001). Given the *in vitro* enzymatic activities of NCER (Shi et al., 2018) and the inverse relationship between the levels of ceramides and *NCER* transcript, the dynamic balance of ceramides in the different developmental stages of BPH are likely regulated by NCER. Consistent with our hypothesis, the role of NCER in the *in vivo* hydrolysis of ceramides was confirmed by the increase in the ceramide levels in BPH whole bodies and ovaries after *NCER* knockdown. Overall, these results suggest that a high level of *NCER* expression is essential in keeping ceramides at low levels in BPH ovaries.

CDases have been shown to play a crucial role by regulating ceramide homeostasis in mammals. A two-fold increase of ceramides was detected in the acid ceramidase gene *Asah1*^±^ in mouse tissues (Li et al., 2002). The molecular and pharmacological inhibition of the neutral ceramidase, encoded by *ASAH2*, increased ceramide levels accompanied by decreased cell survival and increased apoptosis in colon cancer cells (Garcia-Barros et al., 2016). High ceramide levels induced by the ablation of neutral CDase caused a decrease in the number of embryos and an increase of apoptotic cells in zebrafish (Yoshimura et al., 2004). Knocking out *Acer3*, encoding alkaline CDase 3, showed a marked increase of ceramide in the mouse brain, resulting in neuronal degeneration (Wang et al., 2015). In the current study, we found that knocking down *NCER* impaired cellular proliferation and induced apoptosis in BPH ovarioles. This result suggests the role of CDases in regulating ceramide homeostasis and ceramides’ function in inducing apoptosis are evolutionally conserved from insects to mammals. Several studies have shown that ceramides are bioactive, whereas dihydroceramides are inert, and ceramides with different carbon chain lengths have different biological functions. For instance, C16 ceramide is pro-apoptotic, whereas the very long-chain C22:0-C26:0 ceramides are anti-apoptotic (Garic et al., 2019). We propose that since *NCER* knockdown influenced the C20 ceramide more substantially than the other ceramide or dihydroceramide species, NCER likely plays a role in female reproduction by regulating this ceramide. However, the function of specific ceramide or dihydroceramide species still need to be further investigated in insects.

Transmission electron microscopy was conducted to elucidate how NCER regulated BPH ovarian development. TEM analysis showed that *NCER* knockdown disrupted the mitochondrial cristae. Ceramides have been shown to increase mitochondrial outer membrane permeability (MOMP) to initiate apoptosis (Abou-Ghali and Stiban, 2015; Chang et al., 2015; Ueda, 2015). Mitochondria are considered the primary producer of reactive oxygen species (ROS) (Dunn et al., 2015). The dysfunction of mitochondria caused by *NCER* knockdown might trigger a series of cellular damages due to ROS imbalance. Therefore, NCER is vital for maintaining mitochondrial integrity in the BPH ovary. The *NCER* knockdown impairs the female reproductive system by accumulating ceramides, in turn inducing apoptosis and disrupting the mitochondrial structure. The data from the TUNEL analysis also support the above findings by reporting early apoptosis in *NCER*-deficient ovaries. Moreover, the integrity of the mitochondrial membrane structure is critical for the function of mitochondria as a powerhouse of energy metabolism (Melser et al., 2015). With the interruption of the energy metabolism, endocytosis processing in BPH ovarioles will be arrested. Ceramides have been shown to modulate endocytosis by altering the membranes’ biophysical properties (Volpert et al., 2017). In mice astrocytes, the rate of endocytosis of low-density lipoprotein and transferrin was reduced due to the compositional alteration of ceramides with different acyl chains (Volpert et al., 2017). The C6-ceramide decreased the uptake of horseradish peroxidase, the marker of the fluid phase, and inhibited the receptor-mediated endocytosis of low-density lipoproteins in Chinese hamster fibroblasts (Chen et al., 1995). These results suggest that the accumulating ceramides and the mitochondrial damage induced by *NCER* knockdown impair the endocytosis processing in BPH ovarioles, thereby suppress BPH ovarian development.

In conclusion, this study demonstrates that NCER is necessary for the development of the female productive system by controlling ceramide homeostasis in the female ovary. Our study reveals a potential target for developing novel pesticides to control BPH, a highly destructive rice pest.

## Data Availability Statement

The datasets presented in this study can be found in online repositories. The names of the repository/repositories and accession number(s) can be found below: Gene AND XM_022339603.1 and Protein AND ABE28520.

## Author Contributions

CM, Z-RZ, W-WZ, and ChuZ conceived and designed the experiments. X-XS, Z-RZ, and CM wrote the manuscript. CM provided valuable suggestions and revised the manuscript. X-XS, M-FZ, NW, Y-JH, M-JZ, ChaZ, and SA performed the experiments. X-XS and Z-RZ analyzed the data. All authors discussed the results and approved the final manuscript.

## Conflict of Interest

The authors declare that the research was conducted in the absence of any commercial or financial relationships that could be construed as a potential conflict of interest.
