# Supplementary material for: Neutral Ceramidase Is Required for the Reproduction of Brown Planthopper, Nilaparvata lugens (Stål)
Source: Front Physiol. 2021 Feb 24;12:629532. doi: 10.3389/fphys.2021.629532 (PMC7943485; doi:10.3389/fphys.2021.629532)
Supplement: Supplementary file 1 [file Data_Sheet_1.DOCX]

**Neutral ceramidase is required for reproduction of brown planthopper, *Nilaparvata lugens* (Stål)**

Xiao-Xiao Shi^1,2^, Mu-Fei Zhu^1^, Ni Wang^1^, Yuan-Jie Huang^1,4^, Min-Jing Zhang^1^, Chao Zhang^1^, Soomro Abid Ali^1^, Wen-Wu Zhou^1^, Chuanxi Zhang^1^, Cungui Mao^3^ *, Zeng-Rong Zhu^1,2^ *

^1^ State Key Laboratory of Rice Biology; Key Laboratory of Molecular Biology of Crop Pathogens and Insects, Ministry of Agriculture; and Institute of Insect Sciences, Zhejiang University, Hangzhou, Zhejiang, 310058, China;

^2^ Hainan Research Institute, Zhejiang University, Sanya, Hainan, 572024, China;

^3^ Department of Medicine and Stony Brook Cancer Center, The State University of New York at Stony Brook, Stony Brook, New York 11794, USA.

^4^ People’s Government of Fenshui Town, Tonglu County, Hangzhou, Zhejiang, 311519, China

*** Address correspondence to**:

Zeng-Rong Zhu, PhD, Agro-Sciences Building, Institute of Insect Sciences, Zhejiang University, Hangzhou, Zhejiang, 310058, China; Tel./ fax: 0086-(0571)8898-2355; and e-mail: [zrzhu@zju.edu.cn](mailto:zrzhu@zju.edu.cn).

Cungui Mao, PhD, Department of Medicine, 9M0834, MART Building, Stony Brook University, Stony Brook, NY 11794, USA; Tel. 001-(631)216-2904; and e-mail: [cungui.mao@stonybrook.edu](mailto:cungui.mao@stonybrook.edu).


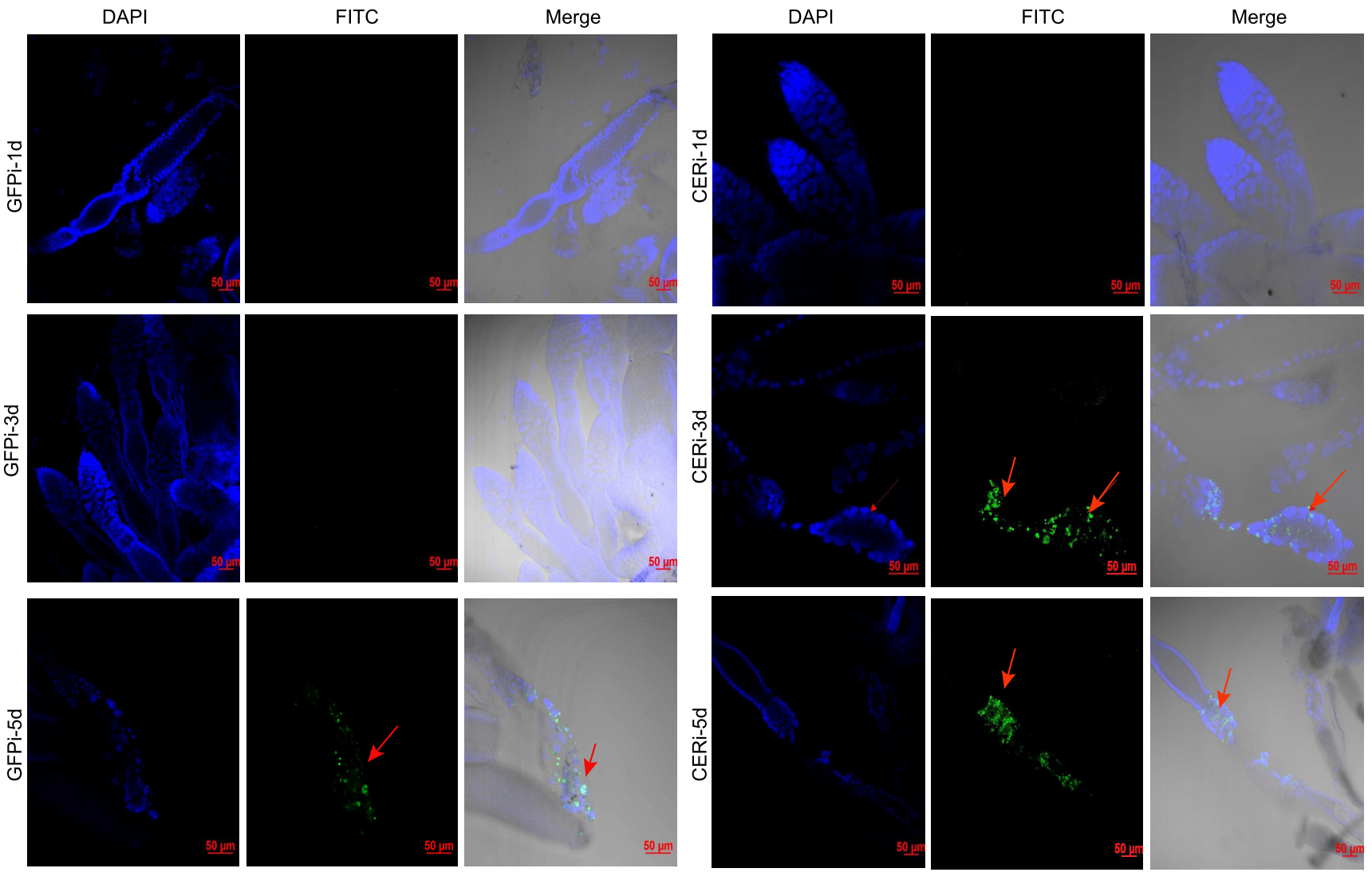


**Supplementary Figure 1. *NCER* knockdown accelerates apoptosis in BPH oocytes**

Ovaries were collected from GFPi or CERi females at 1, 3 or 5 days post injection with dsRNA and subjected to fluorometric TUNEL analysis. The green apoptotic signaling was pointed by red arrow.
